# Particle-Induced Electrostatic Repulsion within an Electric Curtain Operating below the Paschen Limit

**DOI:** 10.3390/mi13020288

**Published:** 2022-02-11

**Authors:** Stuart J. Williams, Joseph D. Schneider, Benjamin C. King, Nicolas G. Green

**Affiliations:** 1 Department of Mechanical Engineering, University of Louisville, Louisville, KY 40292, USA; schneider.devin89@gmail.com (J.D.S.); bcking21@gmail.com (B.C.K.); 2 Electronics and Computer Science, University of Southampton, Southampton SO17 1BJ, UK; ng2@ecs.soton.ac.uk

**Keywords:** electric curtain, electrostatics, dielectrophoresis

## Abstract

The electric curtain is a platform developed to lift and transport charged particles in air. Its premise is the manipulation of charged particles; however, fewer investigations isolate dielectric forces that are observed at lower voltages (i.e., less than the Paschen limit). This work focuses on observations of simultaneous dielectrophoretic and electrostatic forces. The electric curtain was a printed circuit board with interdigitated electrodes (0.020 inch width and spacing) coated with a layer of polypropylene, where a standing wave or travelling wave AC signal was applied (50 Hz) to produce an electric field below the Paschen limit. Soda lime glass beads (180–212 µm) demonstrated oscillatory rolling via dielectrophoretic forces. In addition, several particles simultaneously experienced rapid projectile repulsion, a behavior consistent with electrostatic phenomena. This second result is discussed as a particle-induced local increase in the electric field, with simulations demonstrating that a particle in close proximity to the curtain’s surface produces a local field enhancement of over 2.5 times. The significance of this is that individual particles themselves can trigger electrostatic repulsion in an otherwise dielectric system. These results could be used for advanced applications where particles themselves provided triggered responses, perhaps for selective sorting of micrometer particles in air.

## 1. Introduction

**Figure 1:**
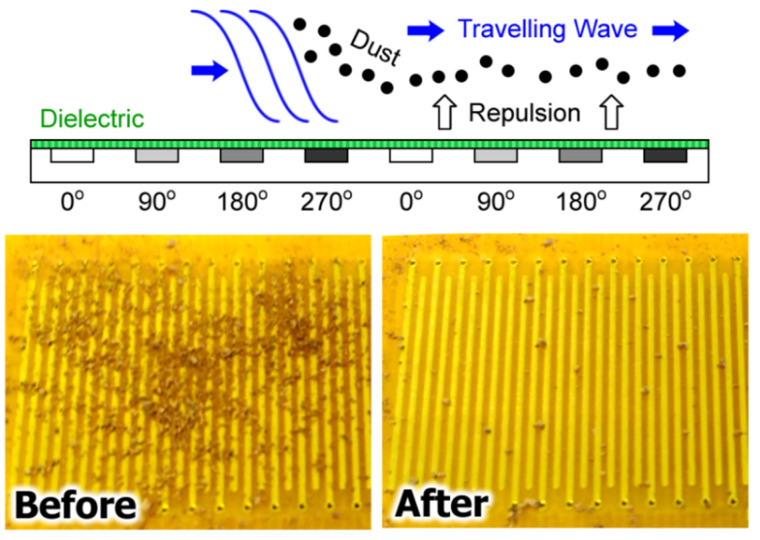
(**top**) Illustration of the electric curtain using four sequentially phase shifted signals at the same frequency applied to successive electrodes in an array. (**bottom**) Experimental results showing the initial and final dust distribution after the application of a 600 V, 50 Hz signal.

The electric curtain is a platform first developed by Masuda [1,2] to lift and transport charged particles, typically in air. The platform itself consists of a series of parallel coplanar electrodes that generate a travelling wave (TW) AC electric field to simultaneously lift particles from the platform surface and translate them away. This multiphysical system is rich in electrokinetic and mechanical physics; particle motion is governed by particle electrokinetic properties (charge and/or induced charge), particle mass (i.e., inertia), field properties (voltage, frequency), platform construction (electrode geometry, dielectric coating), and medium properties (gas composition, pressure, humidity) [3,4,5,6,7]. Figure 1 provides an illustration of a four-phase electric curtain as well as experimental results (using the platform herein) demonstrating the repulsion of dust in ambient conditions using a signal of 600 V and 50 Hz.

Various studies have investigated the electric curtain experimentally [3,8,9,10,11,12,13,14,15,16,17] and with numerical modeling of individual particles’ trajectories [4,6,7,18,19,20,21,22]. The traditional premise of the electric curtain is the manipulation of charged particles via Coulombic forces; it is typically assumed that these particles are either inherently charged or they obtain a net charge from absorption of gaseous ions from corona [3]. The frequency of the applied field coupled with particle inertia [19,20] account for observed oscillatory particle motions. However, seldom are investigations conducted at lower voltages (i.e., in the absence of corona, less than the Paschen limit) where strong repulsion is not typically observed. At these lower voltages dielectrophoretic forces are present but do not contribute towards repulsion, as dielectrophoresis (DEP) is always attractive in air [4,9,22].

Results herein demonstrate that, in the absence of plasma, the particle itself can trigger localized Coulombic repulsion in an otherwise dielectric system. The following provides a brief overview of the theoretical operation of an electric curtain and an introduction to DEP.

### 1.1. Electric Curtain Background

The net force acting on a particle within an electric curtain is typically a combination of Coulombic forces, viscous forces, and gravitational forces. The Coulombic force governs particle translation and is dependent on the net charge of the particle. Standing wave (SW) and TW fields each affect particle translation differently: both repel from the electrode array, but the latter provide lateral translation along the array. Particles can have an inherent charge, or it can be acquired. There are several methods by which a particle can acquire a charge, including tribocharging and corona charging [23]; the latter is associated with traditional electric curtains. Corona-based electrostatic precipitators use macroscale electrodes to generate ionized gas. Similarly, though at the millimeter scale, work by Atten et al. [3] induced particle charging from dielectric barrier discharges where the gaseous ions and electrons are transferred to the particles in the immediate vicinity of the dielectric layer that coats the electrodes. Here the applied voltage needs to be greater than the Paschen voltage, the voltage necessary to start a discharge or electric arc between two electrodes and subsequently ionize surrounding gas.

Inherently, analyzing particle movement within an electric curtain provides a unique challenge, primarily due to the coincidence of the system’s characteristic time and velocity parameters. In most liquid-based electrokinetic microsystems the AC frequency is high (>1 kHz) such that oscillatory electrokinetic effects can be neglected [24] and manipulated colloids reach terminal velocity within milliseconds [25]. However, these characteristics are not shared for low-frequency (<1 kHz) air-based systems, as the applied electrokinetic forces will impart a time-dependent impulsive force leading to a sudden change in particle acceleration. As such, particle mass (i.e., inertia) will be significant [19,20]. Interparticle interactions also occur [4,20], but are outside the scope of this work.

There are three forms of particle translation that have been observed: electric curtain mode [26,27], surfing mode [21], and hopping mode [6]. Electric curtain mode is characterized by high voltages (up to 30 kV) and air ionization. Particles have continuous levitation (balanced with gravity) and translate slower than the wave velocity. Surfing mode occurs with inherently charged particles in an electric field at lower voltages (no corona). Particles move synchronously with the travelling wave in sliding contact with the surface. Hopping mode is produced when charged particles are propelled forward and come to rest on the surface until the wave catches up and initiates the next jump. Interestingly, our results herein do not exhibit these typical translation modes, this is in part due to our operation mode (i.e., no corona). Instead, our findings suggest that particles exhibiting dielectric behavior can subsequently trigger electrostatic repulsion. As such, the following section provides an introduction to relevant dielectric mechanisms.

### 1.2. Dielectric Electrokinetics

A dielectric is a material that can be polarized (i.e., form distinct poles of charge) by an application of an electric field. When a dielectric particle is subjected to a nonuniform electric field DEP occurs. DEP is a well-known particle manipulation technique that has been able to capture, sort, concentrate, and characterize a variety of particles and biological entities as small as a few nanometers [25,28,29]. Unlike electrophoresis, particles do not need to carry a net charge in DEP. The polarization of a particle with DEP is based, in part, on the interfacial polarization at the interface of two dissimilar materials. Particles are either attracted or repelled from regions of greater field strength based on the dielectric properties (permittivity, conductivity) of the particle and the medium it exists in, as well as the applied AC frequency.

For a dipole moment **p** = *q***d**, with opposite charges, *q*, separated by a distance, **d**, subjected to a nonuniform field, **E**, the resultant dielectrophoretic force (neglecting higher order terms) is [25]

(1) F=(p·∇)E.

The dipole moment is given as

(2) $$p=⩝\alpha^{*}E=4\pi \varepsilon_{m}\left(f_{CM}\right)a^{3}E,$$

where ⩝ is particle volume, $\alpha^{*}$ is particle polarizability, *a* is the radius of the particle, ε is permittivity, the subscript *m* refers to the medium. The term *f_CM_* refers to the Clausius-Mossotti factor, defined as

(3) $$f_{CM}=\left(\varepsilon_{p}^{*}-\varepsilon_{m}^{*}\right)/\left(\varepsilon_{p}^{*}+2\varepsilon_{m}^{*}\right),$$

where the subscript *p* refers to particle properties. The complex permittivity $\varepsilon^{*}$ is given by

(4) $$\varepsilon^{*}=\varepsilon -j\sigma /\left(\omega \right),$$

where σ is conductivity, ω=2πf, *f* is the AC frequency, and $j^{2}$ is −1. For a homogeneous spherical particle the dielectrophoretic force, assuming a polarized dipole, is [25,30]

(5) $$F_{DEP}=2\pi \varepsilon_{m}a^{3}Re\left[f_{CM}\right]\nabla E_{rms}^{2}+4\pi \varepsilon_{m}a^{3}Im\left[f_{CM}\right]\left(E_{rms,x}^{2}\nabla \varphi_{x}+E_{rms,y}^{2}\nabla \varphi_{y}+E_{rms,z}^{2}\nabla \varphi_{z}\right).$$

The first term is associated with a standing field, whereas the second term is associated with the phase-dependent portion of the field (φ). In other words, the first component is the traditional dielectrophoretic force and the second is the TW dielectrophoresis (twDEP) term which propels the particle laterally with a translating wave. The magnitude of these forces is greatest at the electrode edge (i.e., location of greatest field strength) and decrease significantly with distance.

The DEP force in Equation (5) assumes that the particle is spherical and homogeneous. Modifications are necessary for multi-shelled and non-spherical particles [31,32]. In addition, as the size of the particle approaches that of the electrode features the dipole approximation needs to be modified to include higher order multipoles (quadropole, etc.) [31,33,34]. Multipoles can increase the dielectrophoretic trapping force significantly [35].

**Figure 2:**
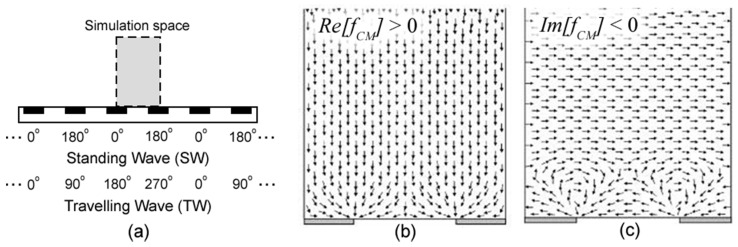
(**a**) Illustration depicting electrode configurations for SW and TW fields. (**b**) Direction of positive DEP force ($Re\left[f_{CM}\right]$ > 0). (**c**) Direction of negative twDEP force ($Im\left[f_{CM}\right]$ < 0). Both (**b**,**c**) are modified from [25].

For particles in air ($\sigma \approx 0,\;\varepsilon_{m}<\varepsilon_{p}$), $Re\left[f_{CM}\right]$ is positive and particles are attracted to greater fields; $Im\left[f_{CM}\right]$ is negative and this force is opposing the direction of the travelling wave. Numerical simulations (Figure 2) show the direction of these DEP force components. Please note that, in air, particles are attracted towards electrode edges in SW fields and would translate against the TW when on the surface.

## 2. Materials and Methods

This manuscript describes several investigations to study the electrokinetics of our electric curtain. (1) First, we could observe dielectrophoretic forces exerted by the curtain using a suspended AFM tip. The AFM would be polarized and actuated due to the applied AC field and dielectric phenomena could be observed. (2) Next, the electrokinetic behavior of Martian dust and spherical particles were captured with a high-speed camera. (3) Last, numerical simulations determined the influence the particle itself had on the local electric field. Details of these investigations follow.

The electric curtain platform used in all experiments consisted of a printed circuit board (ExpressPCB, Santa Barbara, CA, USA) with four sets of repeating interdigitated electrodes (0.020” width and spacing) where a SW (0°–180°–0°–180°) or TW (0°–90°–180°–270°) AC signal was applied. The electrodes were covered with a dielectric of polypropylene tape ($\varepsilon_{r}$ = 2.2–2.36, 0.0016” polypropylene layer, 0.0009” rubber adhesive layer, 76255A21, McMaster-Carr). A four-channel waveform generator (Model 280, Fluke Corp., Everett, WA, USA) sent SW or TW signals to a custom four-channel step-up amplifier which provided potentials up to 800 V_rms_ with frequencies up to approximately 10 kHz. Unless otherwise stated, the applied AC frequency for these tests was 50 Hz.

### 2.1. AFM Experiments

The electrokinetic-induced deflection of a suspended AFM cantilever was measured using an Asylum MFP-3D AFM. The cantilever chip was made of Pyrex and supported two triangle levers. The short lever was 100 µm long × 13.5 µm wide with an approximate stiffness of 0.32 N/m. In each trial, exact cantilever stiffness was calibrated from a force curve on hard silicon surface. The short lever was functionalized with a 20 ± 2 µm diameter borosilicate glass sphere. Before each test, the AFM z-piezo was used to move the lever 34 ± 2 µm away from the surface of the electric curtain. The arm of the cantilever was aligned parallel with the electrodes. Deflection of the cantilever was recorded for two seconds at 10 kHz, while a SW AC signal (50 Hz) was applied to the electric curtain.

### 2.2. Particle Interactions with the Electric Curtain

For initial confirmation of electric curtain behavior, Martian dust simulant (JSC MARS-1A, Orbital Technologies Corp., Madison, WI, USA) was used with a particle diameter of 1 mm and smaller with over 50% of the particles (by weight) had diameters greater than 0.25 mm. The primary chemical composition, by weight, was 34.5% SiO_2_, 18.5% Al_2_O_3_, 9.3% Fe_2_O_3_, and no more than 5% of other individual compounds. A sample of dust was manually applied to the curtain before field activation

Later, experiments were conducted with a second particle type using spherical non-conductive polarizable particles which provided the advantages of consistent particle shape, homogeneity, and size over previous dust samples. Tracking of individual spheres provided additional insight into governing electrokinetic forces. A sparse sample of solid soda lime glass beads (180–212 μm, SLGMS-2.5, Cospheric, Santa Barbara, CA, USA) coated the surface of the electric curtain prior to the activation of a SW or TW field.

A high-speed camera (HiSpec II, 1000 fps) with a zoom lens (Macro 7000, Navitar) acquired videos from both “top view” and “side view” perspectives, showing the interactions and positions of particles relative to the electrodes.

### 2.3. Numerical Simulations

Electrostatics simulations were performed in COMSOL Multiphysics (model details in Supplementary Material) to investigate the influence of the particle position on the local electric field. In brief, the 3D electrostatics system was governed by

(6) $$\varepsilon^{*}=\varepsilon -j\sigma /\left(\omega \right),$$

where *V* is electric potential and *ρ* is volume charge density. For simplicity, we assumed negligible volume charge (*ρ* = 0) and used *ε_r_* = 1 for air, *ε_r_* = 7 for the glass particle, and *ε_r_* = 2.28 for the insulative layer. Here, *ε* = *ε_r_ε_o_* with *ε_r_* as the relative permittivity and *ε_o_* the permittivity of free space. A spherical particle was placed over an electrode edge at various heights above the insulator layer (0.5 µm to 300 µm) and the resulting local electric field was modeled.

## 3. Results and Discussion

### 3.1. AFM Experiments

**Figure 3:**
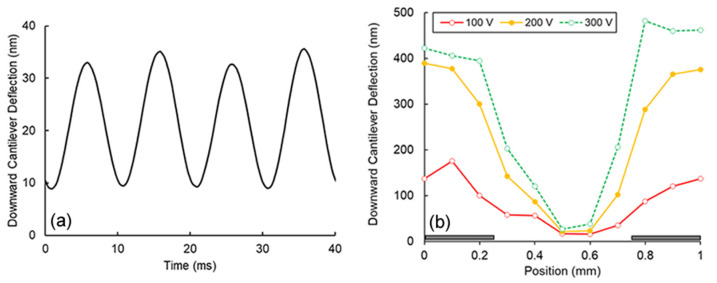
(**a**) A portion of the time trial demonstrating oscillatory deflection of an AFM for a 50 Hz field. (**b**) AFM deflection as a function of voltage and position. Electrodes are illustrated as dark rectangles.

Figure 3a shows the oscillation of the cantilever deflection for a 50 Hz, 300 V signal for a 40 ms period. The cantilever position was approximately centered between two electrode strips. The oscillation period was 10 ms (i.e., 100 Hz), twice that of the applied AC frequency—this characteristic is evidence of induced dielectric forces. However, electrostatic forces were not negligible. The amplitude of every other wave was measurably greater than the preceding peak, suggesting a non-neutral charge on the suspended AFM. Figure 3b shows the downward attractive deflection of the cantilever as a function of applied voltage (100 V–300 V, 50 Hz). The results qualitatively agree with the expected SW DEP force being greatest near the electrode edges and increase with applied voltage (Figure 2b). Additional tests without a tip (cantilever only) also demonstrated similar deflection behavior suggesting that the cantilever itself was also polarized (results not shown); therefore, the magnitude of the resultant measured attractive force was not due solely by its attached glass sphere. Qualitatively, these AFM results demonstrated that dielectric forces exist for objects not in direct contact with the surface of the electric curtain. Furthermore, this setup is not too far removed from Pohl and Pethig’s apparatus [36] that demonstrated positive dielectrophoresis of a suspended object in air. In the future, the use of optical tweezers [37] could be used to decouple any mechanical attachments to the suspended particle under investigation.

### 3.2. Particle Interactions with the Electric Curtain

Dust was successfully repelled from the surface of an electric curtain platform using an applied voltage of 600 V at 50 Hz for both SW and TW fields (Figure 1, bottom), though the latter demonstrated repulsion in the direction of the translating wave. Dust repulsion was frequency dependent; at AC frequencies greater than 1 kHz, there was no observable particle movement. More specifically, for frequencies between 230 Hz and 1 kHz, limited particle ‘agitation’ was observed, consistent with previous observations [3], though no bulk repulsion occurred. However, once the AC frequency decreased to approximately 210 Hz there was sudden and immediate particle repulsion; repulsion occurred for all tested frequencies below this limit (10 Hz to 210 Hz). Several larger particles whose size was similar to the electrode gap were trapped in the dielectric-coated regions (Figure 1, bottom-right). This further suggests dipole (and likely multipole [33,34]) generation, subsequent electro-orientation (the alignment of the particle’s long axis in the direction of the field [31]), and demonstrates single particle dielectrophoretic trapping.

High-speed videos (1000 fps) were acquired from a “side view” in order to observe bulk dust repulsion behavior. The video showed that dust would be repelled from the surface in a pulse-like manner whose period (10 ms) corresponded to twice the applied frequency (Supplemental Material, Video S1). These results were consistent with the AFM experiments in which the applied forces occur at twice the applied AC frequency, suggesting dielectric behavior. However, particles experiencing DEP in should be attractive, as demonstrated by the AFM results. This observed discrepancy of repulsive behavior motivated the proceeding study using homogeneous spherical glass particles of consistent size.

**Figure 4:**
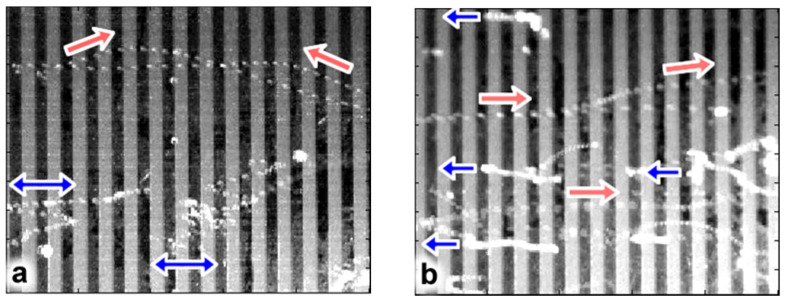
Consecutive overlaid images over a 0.2 s interval (2 ms between images) for both SW and TW fields. (**a**) SW manipulation of glass spheres where both oscillatory rolling (blue arrows) and high velocity repulsion (red arrows) were observed. (**b**) TW manipulation where particles would roll slowly against the field (blue arrows) or be repelled rapidly in the direction of the applied field (red arrows).

At 50 Hz and 600 V, spherical glass particles were repelled for both SW and TW fields. For both scenarios particles experienced low velocity oscillatory “rolling” along the surface and/or rapid repulsive projectile motion. For an SW field, rolling particles would typically oscillate about an electrode strip and about its nearest electrode neighbors (Figure 4a, blue double arrow). This behavior suggests particles experienced positive DEP and, when including inertial effects, the oscillatory rolling particle motion (blue arrows) makes sense. However, repulsed projectile particles (Figure 4a, red arrow) were observed at velocities much greater than the oscillatory velocity. These particles initiated their “launch” in close proximity to electrode edges (where the field is strongest) and projection occurred in approximately 10 ms intervals (twice the applied AC frequency).

TW results also demonstrated particle rolling and projectile motion though with specific, and opposite, directionality. The particles rolled against the TW field direction relatively slowly (Figure 4b, blue arrows), while others were projected at high velocity in the direction of the wave (Figure 4b, red arrows). Due to the negative $Im\left[f_{CM}\right]$ at 50 Hz and the direction of the twDEP force near the electrode surface (Figure 2c), dielectrophoretic forces are responsible for each particle’s roll direction that is opposite of the TW. Particle repulsion in the direction of the TW field implies electrostatic repulsion and is consistent with typical electric curtain performance. High speed videos of both “top view” and “side view” SW and TW experiments are available in Supplementary Materials (SW: Videos S2 and S3; TW: Videos S4 and S5).

For both SW and TW studies, the projectile motion of particles was initiated near the electrode edge where the electric field is greatest. However, in the absence of particles, arching nor plasma was not observed, even when the voltage increased to the upper limit of our equipment (800 V). From these results it was hypothesized that the particles themselves triggered local electrostatic repulsion. As DEP attracted particles towards regions of greater field strength, the particle itself would locally distort the field further, intensifying it to the point of triggering electrostatic repulsion. Thus, the following numerical simulations were conducted to determine the significance of field amplification due to the presence of the particle itself.

### 3.3. Numerical Simulations

**Figure 5:**
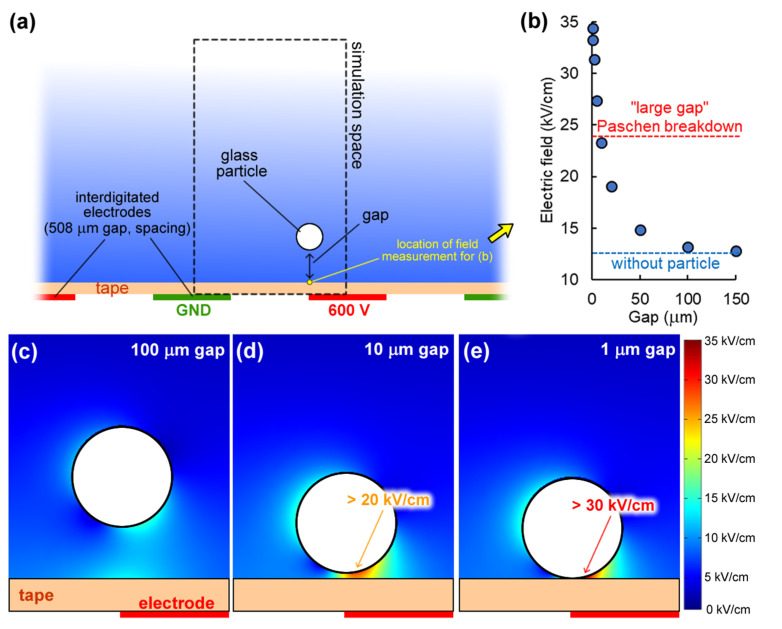
(**a**) Depiction of the simulation space (more details in Supplementary Material). (**b**) Magnitude of electric field at the indicated measurement point. As the gap decreases the localized electric field increases. (**c**) Numerical simulation result of the electric field magnitude near the particle for a gap of 100 µm; similarly for (**d**) 10 µm and (**e**) 1 µm gaps.

Figure 5a depicts the cross-section view of the 3D electrostatics model and the simulated space (more details in Supplementary Material). The sphere was placed centered with the electrode edge and its gap above the insulator was varied. The electric field magnitude at the point directly above the electrode edge on the insulator layer was measured (Figure 5a,b). In the absence of a particle, the electric field at this location was modeled to be 12.5 kV/cm and it did not significantly increase until the gap between the particle and insulator layer was less than one particle diameter. Figure 5c, Figure 5d and Figure 5e show a zoomed-in portion of the simulation for gaps of 100 μm, 10 μm, and 1 μm, respectively. The space between the particle and the insulative layer experiences a significant increase in electric field, such that the modeled 0.5 μm gap was 34.4 kV/cm (2.8 times greater than the modeled field without the particle).

Numerical simulations definitively show that particles themselves significantly increase the electric field locally. Next, these modeled values were compared to Paschen’s law, which is the breakdown voltage where a discharge would occur between two electrodes as a function of gap length [38]. For a uniform field and “very large gaps”, the limiting value of the field under normal temperature and pressure is approximately 24 kV/cm (labeled in Figure 5b). It is unlikely that the field limit for our electric curtain system occurs at 24 kV/cm, as our electrodes are insulated and planar, as the breakdown voltage would differ for non-uniform fields [39]. Although we do not know the breakdown voltage for our electric curtain, the experimental and numerical evidence herein supports that particle-induced electrostatic repulsion is a reasonable phenomenological explanation of the observed behavior.

## 4. Concluding Remarks

Positive dielectrophoretic forces were expected [36] for our experimental conditions (i.e., without corona); however, simultaneous Coulombic repulsion was unexpected until we accounted for local particle-induced field distortions. These experiments demonstrate an interesting electrokinetic behavior where particles exhibit dipolar (DEP, “pulsed” repulsion at twice the applied frequency) whose attractive forces distort the electric field and subsequently trigger monopolar (Coulombic repulsion) forces. These findings serve as a guide for future work in investigating the interaction between particles and the electric field in electric curtains.

By no means is this experimental study comprehensive, as charge transfer mechanisms are complex. For example, work by J. Lowell demonstrated that charge transfer between two insulators can occur without tribocharging [40]. Furthermore, Lowell showed that polarization significantly influenced charge transfer energy states [41], regardless of whether the states are donors or accepters. Furthermore, particle and insulative layer polarizability is frequency dependent, and this characteristic has been explored previously where directionality of particle motion in a TW curtain was AC frequency dependent [20]. Generally, particles would translate with the wave at low frequencies but would move in the opposite direction at higher frequencies; at even greater frequencies no net particle movement would occur. Thus, understanding frequency-dependent polarizability within a dielectric curtain should be further explored in applying this technology towards particle sorting.

## Data Availability

Videos are available in Supplementary Material. AFM data are available upon request from the corresponding author, S.J.W.

## Supplementary Materials

The following supporting information can be downloaded at https://www.mdpi.com/article/10.3390/mi13020288/s1. A document describing the COMSOL Multiphysics numerical simulations and a table of results that was plotted in Figure 5b (Figures S1 and S2, and Table S1). A high-speed video of pulsed repulsion of dust is available (Video S1). High speed “top view” and “side view” videos of soda lime glass repulsion are available for SW configuration (Videos S2 and S3) and TW configuration (Videos S4 and S5).
