# Supplementary material for: Particle-Induced Electrostatic Repulsion within an Electric Curtain Operating below the Paschen Limit
Source: Micromachines (Basel). 2022 Feb 11;13(2):288. doi: 10.3390/mi13020288 (PMC8878500; doi:10.3390/mi13020288)
Supplement: Supplementary file 1 [file micromachines-13-00288-s001.zip › micromachines-1567579-supplementary.pdf]

Particle-induced electrostatic repulsion within an electric curtain operating below the Paschen limit

Description of Videos

Video S1: This is a side view high speed video (1,000 fps) to observe dust repulsion with an applied signal of 600 V at 50 Hz for a SW system. The video showed that dust would be repelled from the surface in a pulse-like manner whose period (10 ms) corresponded to twice the applied frequency.

Video S2: This is a side view high speed video (1,000 fps) observing the behavior of spherical glass particles at 600 V and 50 Hz for a SW system.

Video S3: This is a top view high speed video (1,000 fps) observing the behavior of spherical glass particles at 600 V and 50 Hz for a SW system.

Video S4: This is a side view high speed video (1,000 fps) observing the behavior of spherical glass particles at 600 V and 50 Hz for a TW system. The travelling wave field goes from left to right.

Video S5: This is a top view high speed video (1,000 fps) observing the behavior of spherical glass particles at 600 V and 50 Hz for a TW system. The travelling wave field goes from left to right.

Numerical Simulation

A 3D numerical simulation was conducted in COMSOL Multiphysics using their "Electrostatics" module. The subdomain of this module is governed by

$$-\nabla \cdot (\varepsilon \, \nabla V) = \rho \qquad \text{(S1)}$$

where V is electric potential and ρ is volume charge density, and ε is the permittivity of the material. Here, $\varepsilon = \varepsilon_r \varepsilon_0$ with ε_r as the relative permittivity and ε_0 the permittivity of free space. For simplicity, we assumed negligible volume charge ($\rho = 0$) and used $\varepsilon_r = 1$ for air, $\varepsilon_r = 7$ for the glass particle, and $\varepsilon_r = 2.28$ for the insulative layer.

Due to the symmetric nature of the interdigitated electrode geometry, only a fraction of the system needed to be modeled–this is illustrated in Figure S1. Half of each electrode pair was included in the model and were set as ground ($V = 0$, i.e., a Dirichlet boundary condition) or at the applied potential ($V = 600$ V)–these are colored blue in Figure S1. The other boundary conditions of the model were set as insulative ($\partial V / \partial n = 0$, i.e., a Neumann boundary condition)–these are colored green in Figure S1.

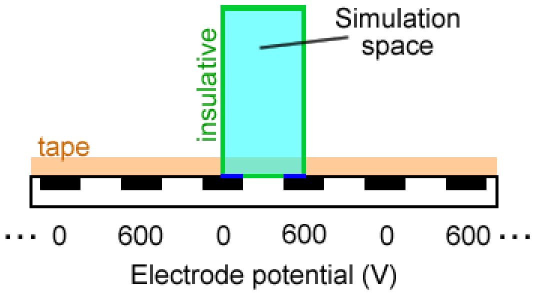

Figure S1. Depiction of the simplified numerical simulation space due to interdigitated electrode symmetry.

The following will discuss the dimensions of the system which are depicted in Figure S2. The electrode width and spacing was 0.020 inches, or 508 µm. The insulator tape layer thickness was 63.5 µm. The footprint of the simulation was 2.0 mm wide and 5.0 mm in height. The diameter of the spherical glass particle was 196 µm. The particle was placed over an electrode edge at various heights above the insulator layer (0.5 µm to 300 µm).

The electric field directly below the particle on the insulator surface was measured and plotted in Figure 5b and the numerical results are shown in Table S1.

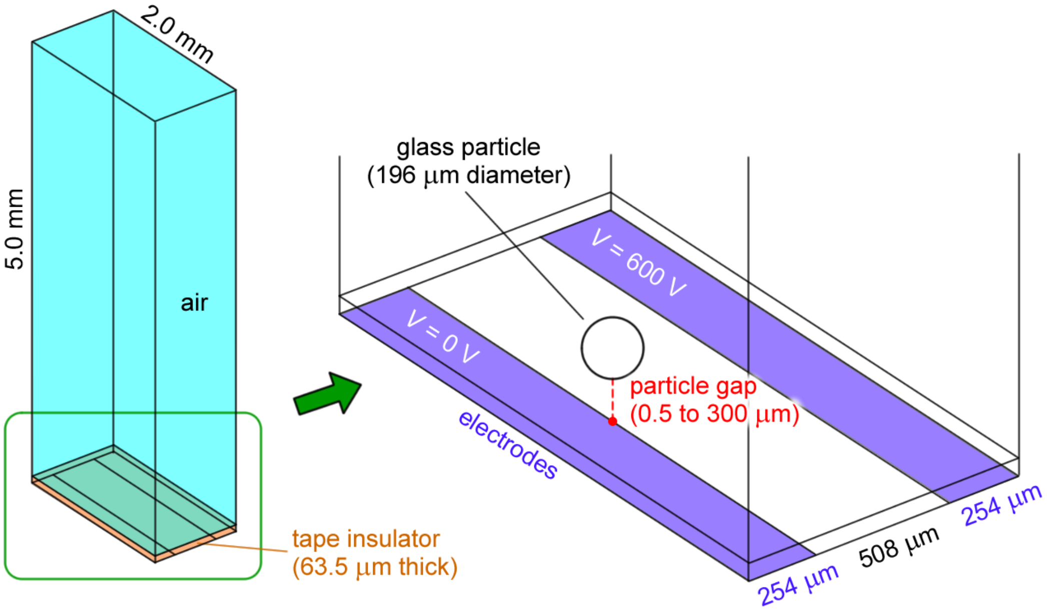

Figure S2. Dimensions of the numerical simulation space.

Table S1. Results of the numerical simulation at the point of interest for various particle heights.

Particle gap (μm).	Electric field (kV/cm)..
0.5.	34.41..
1.	33.28..
2.	31.41..
5.	27.36..
10.	23.32..
20.	19.10..
50.	14.81..
100.	13.19..
150.	12.78..
200.	12.62..
250.	12.55..
300.	12.51..
no particle.	12.46..
